# Implementation of Whole-Body MRI (MY-RADS) within the OPTIMUM/MUKnine multi-centre clinical trial for patients with myeloma

**DOI:** 10.1186/s13244-022-01253-0

**Published:** 2022-07-28

**Authors:** Mihaela Rata, Matthew Blackledge, Erica Scurr, Jessica Winfield, Dow-Mu Koh, Alina Dragan, Antonio Candito, Alexander King, Winston Rennie, Suchi Gaba, Priya Suresh, Paul Malcolm, Amy Davis, Anjumara Nilak, Aarti Shah, Sanjay Gandhi, Mauro Albrizio, Arnold Drury, Sadie Roberts, Matthew Jenner, Sarah Brown, Martin Kaiser, Christina Messiou

**Affiliations:** 1 Royal Marsden NHS Foundation Trust and Institute of Cancer Research, Downs Road, SM2 5PT, Sutton, London, UK; 2 University Hospital Southampton NHS Foundation Trust, Southampton, UK; 3 Leicester Royal Infirmary, Leicester, UK; 4 Royal Stoke University Hospital, Stoke-on-Trent, UK; 5 University Hospitals Plymouth NHS Foundation Trust, Plymouth, UK; 6 Norfolk and Norwich University Hospital, Norwich, UK; 7 Epsom and St. Helier University Hospitals NHS Trust, Epsom, UK; 8 Worcestershire Acute Hospitals NHS Trust, Worcester, UK; 9 Basingstoke and North Hampshire Hospital, Basingstoke, UK; 10 North Bristol NHS Trust, Bristol, UK; 11 Nottingham University Hospitals NHS Trust, Nottingham, UK; 12 Royal Bournemouth and Christchurch Hospitals NHS Foundation Trust, Bournemouth, UK; 13 University of Leeds Clinical Trial Research Unit, Leeds, UK

**Keywords:** Whole-body MRI, DWI, T1-w Dixon, Myeloma, Multi-centre clinical trial

## Abstract

**Background:**

Whole-body (WB) MRI, which includes diffusion-weighted imaging (DWI) and T_1_-w Dixon, permits sensitive detection of marrow disease in addition to qualitative and quantitative measurements of disease and response to treatment of bone marrow. We report on the first study to embed standardised WB-MRI within a prospective, multi-centre myeloma clinical trial (IMAGIMM trial, sub-study of OPTIMUM/MUKnine) to explore the use of WB-MRI to detect minimal residual disease after treatment.

**Methods:**

The standardised MY-RADS WB-MRI protocol was set up on a local 1.5 T scanner. An imaging manual describing the MR protocol, quality assurance/control procedures and data transfer was produced and provided to sites. For non-identical scanners (different vendor or magnet strength), site visits from our physics team were organised to support protocol optimisation. The site qualification process included review of phantom and volunteer data acquired at each site and a teleconference to brief the multidisciplinary team. Image quality of initial patients at each site was assessed.

**Results:**

WB-MRI was successfully set up at 12 UK sites involving 3 vendor systems and two field strengths. Four main protocols (1.5 T Siemens, 3 T Siemens, 1.5 T Philips and 3 T GE scanners) were generated. Scanner limitations (hardware and software) and scanning time constraint required protocol modifications for 4 sites. Nevertheless, shared methodology and imaging protocols enabled other centres to obtain images suitable for qualitative and quantitative analysis.

**Conclusions:**

Standardised WB-MRI protocols can be implemented and supported in prospective multi-centre clinical trials.

*Trial registration* NCT 03188172 clinicaltrials.gov; registration date 15th June 2017 https://clinicaltrials.gov/ct2/show/study/NCT03188172

## Key points

- WB-MRI is embedded in guidance (from the International Myeloma Working group) for imaging patients with myeloma.
- WB-MRI, which includes diffusion-weighted imaging and T_1_-w Dixon, permits qualitative and quantitative measurements of bone marrow.
- A WB-MRI protocol was successfully implemented at 12 sites (3 vendors/2 field strengths).
- MY-RADS standardised WB-MRI protocols can be successfully implemented in prospective multi-centre clinical trials.

## Background

Advances in outcome-improving therapies for myeloma patients have driven the need for sensitive detection of focal lesions in the bone marrow. The International Myeloma Working Group (IMWG) has updated the long-standing diagnostic imaging criteria from detection of cortical bone destruction on plain film/CT to now include the option to treat based on a positive MRI [1]. The shift towards earlier disease detection in the marrow prior to cortical bone destruction is based on evidence that, if disease is detected early, and patients are treated according to risk, then survival advantages are conferred [2–9]. The high sensitivity of whole-body magnetic resonance imaging (WB-MRI) for detecting focal marrow lesions is explicitly acknowledged [10] and MRI is described as the “gold standard” for imaging the axial skeleton [11]. In one prospective study [12], WB-MRI helped to identify at least one focal lesion in 83% of participants compared to 60% by ^18^F Fluorodeoxyglucose PET/CT (FDG PET/CT). In addition, diffuse disease was detected in 82% of participants compared with 17% with FDG PET/CT. However, limited availability of WB-MRI globally has led to pragmatic guidance from the IMWG, which suggests that low-dose whole-body CT can be used first-line, reserving WB-MRI for instances where the CT is negative or equivocal [11, 13–17]. Nonetheless, national guidance in the UK positions WB-MRI as first-line imaging for all patients with suspected myeloma, based not only on accuracy, but also on longer-term impact on quality of life and health economics [18].

Contemporary WB-MRI protocols include both anatomical and functional MR sequences and their complementary role strengthens the detection of disease and therapy response assessment when compared to anatomical sequences only. The high sensitivity of WB-MRI has also seen its role emerge for restaging, where assessment of the marrow space becomes of even greater relevance, as cortical bone defects from previously treated disease can remain stable for many years despite evolving marrow disease activity. Contemporary WB-MRI, which includes functional diffusion-weighted MRI (DW-MRI) and T_1_-weighted (T_1_-w) Dixon MRI, has not only scaled up sensitivity but also permits quantitative measurements of both disease and response to treatment [19]. In particular, the advent of DW-MRI allows differentiation of focal active sites from treated inactive sites. The inability of CT to reliably detect small volume disease in the marrow space and to differentiate treated from active sites of disease has led to its exclusion from IMWG recommendations for imaging residual disease [20]. The advanced capabilities of WB-MRI naturally lend themselves to supporting clinical decision making where sensitivity for detection of disease is paramount, such as detection of Minimal Residual Disease (MRD) following marrow transplantation. To date, evidence and guidelines support the role of fluorodeoxyglucose (FDG) PET/CT in this setting [20, 21] whilst emerging evidence for WB-MRI looks promising [22–24].

The addition of WB-MRI to international guidance and evolution and expansion of WB-MRI capabilities created the need for standardisation of acquisition and reporting which was established by the MY-RADS international consensus [10]. Applying the MY-RADS criteria, Belotti et al. [24] were able to show superior overall survival in patients with complete imaging response.

However, implementation of WB-MRI for multi-centre clinical trials and routine clinical use in myeloma requires centres to set up high-quality imaging protocols, which must be adapted to the diverse range of MRI hardware and software present in clinical use. MY-RADS recommendations, and other similar WB-MRI protocol recommendations such as MET-RADS-P [25] and ONCO-RADS [26], mandate key imaging protocol parameters such as coverage, slice thickness, orientation and *b*-values for DW-MRI, but do not specify values for all imaging protocol parameters; centres must, therefore, optimise these other parameters to achieve high-quality images.

Development of robust imaging protocols is challenging, particularly for DW-MRI, as a plurality of parameters must be optimised to achieve sufficient signal-to-noise ratio (SNR), acceptable geometric integrity and limited image ghosting, whilst ensuring that total scan time is tolerable. Optimal selection of these protocol parameters, for example the choice of diffusion encoding scheme, the number of image averages and receiver bandwidth, may vary between hardware and software versions, though some broad recommendations are available [27].

The requirement for further optimisation of imaging protocols, in addition to the key parameters specified in WB-MRI recommendations, and the variation between MRI hardware and software versions, creates a need for further guidance in WB-MRI protocol optimisation to facilitate adoption across a larger number of centres. Some centres may lack the resources and experience to develop their own WB-MRI protocols and may prefer to implement validated protocols that have been developed by experienced centres. This is especially important in the context of multi-centre trials where MR-derived imaging biomarkers (average Apparent Diffusion Coefficient, ADC, or tumour volume for example [28]) act as surrogate endpoints for treatment response and/or patient stratification; it is essential that protocols are harmonised across participating institutions to ensure that results can be collectively analysed. Standardisation of the ADC biomarker has already been published by the Quantitative Imaging Biomarkers Alliance [29] for several organs (brain, prostate and liver), but unfortunately no such recommendations are available yet for WB scans.

We report on the first imaging sub-study (IMAGIMM, IMAGIng Minimal residual disease in Myeloma) to embed standardised WB-MRI within a prospective, UK multi-centre myeloma clinical trial (OPTIMUM/MUK nine; clinicaltrials.gov NCT03188172) in order to explore the use of WB-MRI to detect treatment effect. This work describes the development of the imaging sub-study setting up, in order to obtain high-quality images for qualitative and quantitative analysis. Moreover, by sharing this methodology and our imaging protocols, we hope to enable easier implementation of WB-MRI at other centres and to facilitate future WB-MRI research, with a reduced burden on time and resources at each site.

## Methods

### Protocol harmonisation

Based on previous work [19], a standardised WB-MRI protocol compliant with the MY-RADS [10] was set up locally on a 1.5 T scanner (Aera, Siemens Healthineers, Erlangen, Germany) at the lead site. An imaging manual describing the suggested MR protocol, the quality assurance (QA)/quality control (QC) procedures and data transfer was produced and provided to the other 11 participating sites. The available fleet of scanners involved in this multi-centre imaging trial is presented in Table 1.

**Table 1:** Trial participating scanners across the 12 sites

Sites	Scanner information				
	Vendor	Model	Magnetic field	Software	WB-MRI Protocol
**1**	**Siemens**	**Aera**	**1.5 T**	**VE11C**	**protocol A**
2	Siemens	Aera	1.5 T	VE11C	protocol A
**3**	**Siemens**	**Skyra**	**3 T**	**VE11C**	**protocol B**
4	Siemens	Aera	1.5 T	VE11C	protocol A
5	Philips	Ingenia	1.5 T	5.4.1	protocol C
**6**	**GE**	**Discovery**	**3 T**	**DV26**	**protocol D**
7	Siemens	Aera	1.5 T	VE11C	protocol A
**8**	**Philips**	**Ingenia**	**1.5 T**	**5.4.1**	**protocol C**
9	Siemens	Aera*	1.5 T	VE11C	protocol A
10	Philips	Ingenia	1.5 T	5.3.1	protocol C
11	Siemens	Aera	1.5 T	VE11C	protocol A
12	Siemens	Avanto/Aera	1.5 T	VE11C	protocol A

A = protocol site 1 = Siemens Aera 1.5 T; 7 sites

B = protocol site 3 = Siemens Skyra 3 T; 1 site

C = protocol site 8 = Philips Ingenia 1.5 T; 3 sites

D = protocol site 6 = GE 3 T; 1 site

*scanner with lower hardware specifications

The full MR protocol included 5 sequences: survey, multi-station axial DW-MRI (vertex to knee), multi-station axial T_1_-w Dixon (vertex to knee) and sagittal T_1_-w and T_2_-w of the whole spine. Multi-station axial T_2_-w sequence was not included (optional as per MY-RADS guidance) to allow for a total imaging time of typically less than one hour. Where possible, essential MR parameters of the functional sequences (DWI and T_1_-w Dixon) such as in-plane resolution, slice thickness, field of view and *b*-values (for DWI) were kept constant across scanners to ensure a harmonised multi-site protocol. The work presented here concentrated on the functional WB-MRI sequences only, as these two sequences are essential for facilitating future quantitative WB-MRI research and are less familiar to sites within WB-MRI protocols.

### Data acquisition

**Fig. 1:**
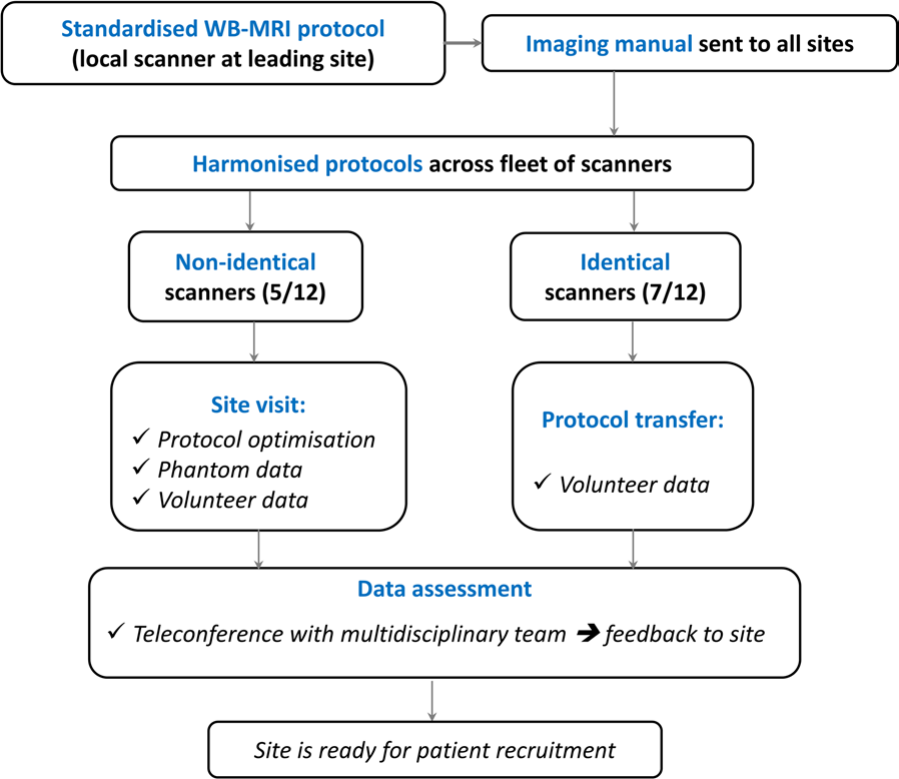
Schematic of site set-up across the participating 12 sites.

Two approaches were considered depending on the scanner type and the site’s previous experience with WB-MRI (see schematic in Fig. 1), whilst allowing for protocol harmonisation.

The first approach involved personal site visits by the physics team from the lead site to support protocol set-up at sites that had non-identical scanners (different vendor or magnetic field strength compared with the lead site’s scanner). Two such visits were organised for the development of protocols B (site 3) and C (site 8), see Table 1. Unfortunately, due to time limitation, no visit was performed for protocol D (site 6) and harmonisation of the locally available protocol was instead considered.

Each visit was typically 4 h and covered protocol optimisation, followed by data acquisition using the developed protocol on two test objects and on one healthy volunteer. Two home-made test objects were used to assess quality of the ADC quantification for DW-MRI and fat/water separation for T_1_-w Dixon. First, a temperature equilibrated ice-water phantom (preparation and procedures detailed in [30]) assessed accuracy of the apparent diffusion coefficient (ADC) at a set temperature of 0 °C within 5 vials with known ADC properties. The composition of each vial was as detailed below: vial 1 (0% sucrose and 0.13 mM MnCl_2_), vial 2 (0% sucrose and 0.0 mM MnCl_2_, i.e. pure water), vial 3 (10% sucrose and 0.087 mM MnCl_2_), vial 4 (10% sucrose and 0.0 mM MnCl_2_) and vial 5 (20% sucrose and 0.065 mM MnCl_2_).

Second, a two-compartment fat–water phantom consisting of concentric cylinders of doped water and corn oil [31] assessed both quality of fat suppression and homogeneity of ADC estimation within an imaging station along the cranio-caudal direction (within the homogenous doped water compartment). Quantitative results from the test objects have previously been assessed in a multi-centre clinical trial [30] and a multi-vendor, multi-field-strength imaging study [31], respectively.

The second approach was adopted for sites that used identical scanners to the ones already approved at the lead site or other visited sites. This faster approach comprised sending the scanner-specific imaging protocol in a format that can be directly imported onto the site’s scanner and was followed by image acquisition in a healthy volunteer by the local team. That covered protocols A and C, specific to Siemens Aera 1.5 T and Philips Ingenia 1.5 T scanners, respectively.

The four main MR protocols developed for the multi-centre imaging sub-study are detailed in Table 2.

**Table 2:** WB-MRI protocol (DWI and T_1_-w Dixon) for four main scanners across the 12 sites

MRI Parameters	DWI				T_1_-w Dixon			
Sequences	2D single-shot echo planar imaging				3D gradient echo			
Scanner type	1.5 T Siemens	1.5 T Philips	3 T Siemens	3 T GE	1.5 T Siemens	1.5 T Philips	3 T Siemens	3 T GE
Protocol Labelling	A	C	B	D	A	C	B	D
Vendor sequence name	DWI	DWI	DWI	DWI	Vibe Dixon	FFE mDixon	Vibe Dixon	Lava Flex
Acquisition plane	Axial	Axial	Axial	Axial	Axial	Axial	Axial	Axial
Breathing mode^1^ (FB/BH)	FB	FB	FB	FB	BH	BH	BH	BH
Station acquisition time [min:s]	04:47	04:21	03:37	05:13	00:17	00:17	00:14	00:19
Number of averages (per b value)	3, 6, 6	3, 6, 6	3, 6, 6	3, 6, 6	1	1	1	1
Acquired resolution [mm^2^]	3.2 × 3.2	3.2 × 3.2	3.2 × 3.2	3.2 × 3.2	1.6 × 1.6	1.6 × 1.6	1.6 × 1.6	1.6 × 1.6
Reconstructed resolution [mm^2^]	1.6 × 1.6	1.6 × 1.6	1.6 × 1.6	3.2 × 3.2	0.8 × 0.8	0.8 × 0.8	0.8 × 0.8	0.8 × 0.8
Slice thickness [mm]	5	5	5	5	5	5	5	5
Slices per station	40	40	40	36	40	40	52	40
Slice gap [mm]	0	0	0	0	0	0	0	0
Slice oversampling [%]	–	–	–	–	20	27.9	23.1	na
TR [ms]	6240	5664	4690	6000	7.6	5.3	4.2	5.4
TE [ms]	73	75	71	66	2.39/4.77	1.73/3.6	1.23/2.46	1.2/2.4
TI [ms] (STIR^2^ fat suppresion)	180	180	260	260	–	–	–	–
Flip angle [°]	90	90	90	90	16	16	16	12
Acquisition matrix (read dir.)	134	136	132	134	256	268	256	276
FOV (read direction) [mm]	430	430	430	430	430	430	430	430
FOV (phase direction) [%]	81	83	82	100	75	75	75	100
Receiver bandwidth^3^ [Hz/Px]	1964	3025 [water–fat shift 5.6 pixel]	1994	3732 [± 250 kHz]	400	676 [water–fat shift 0.3 pixel]	1030	1208 [± 166.67 kHz]
Parallel imaging^4^/acceleration	GRAPPA 2 (30 ref. lines)	SENSE 2.5	GRAPPA 2 (30 ref. lines)	ASSET 2	CAIPIRINHA 2 × 2	SENSE (phase reduction) 2.5	CAIPIRIHNA 2 × 2	ARC (phase reduction) 2
Phase partial Fourier	7/8	no	7/8	–	no	no	no	no
Diffusion mode^5^	3-Scan Trace	Grad. overplus	4-Scan Trace	ALL (3 in 1)	–	–	–	–
Diffusion encoding scheme	bipolar	monopolar	monopolar	monopolar	–	–	–	–
3 *b*-values [s/mm^2^]	50, 600, 900	50, 600, 900	50, 600, 900	50, 600, 900	–	–	–	–
Station number	6	6	6	6	7	7	7	7
Station overlap [mm]	0	0	0	0	20	20	60	20

^1^FB free breathing; BH breath holding.

^2^STIR short Tau inversion recovery; TI inversion time

^3^Receiver bandwidth is quoted in Hz/Px for all manufacturers; For Philips and GE manufacturers their equivalent terminology is presented in square brakets

^4^GRAPPA Generalised autocalibrating partially parallel acquisition; SENSE sensitivity encoding; ASSET array spatial sensitivity encoding technique; CAIPIRINHA Controlled Aliasing in Parallel Imaging Results in Higher Acceleration; ARC Autocalibrating Reconstruction for Cartesian imaging

^5^Three-scan trace and gradient overplus use three mutually orthogonal diffusion gradient directions (not aligned with the cardinal directions of the scanner); ALL uses 3 gradient directions aligned with scanner's directions. Four-scan trace use four diffusion gradient directions. In all cases, images from 3, or 4, respectively, diffusion gradient directions were combined to produce trace images

### Data archive

Post-processing steps were tested at each participating site using the acquired volunteer data, including: (i) calculation of ADC maps from DW-MRI (using 3 *b*-value images), (ii) calculation of fat- and water-only images from T_1_-w Dixon images, and (iii) composition of imaging station data into full-body series (one series per *b*-value, and three series for ADC maps, fat-only images and water-only images, respectively). These images were further anonymised on-site prior to data transfer. A complete anonymised imaging dataset from a healthy volunteer or patient comprises both data acquired at each individual imaging station and, where possible, the automatically composed images across all stations. Anonymised data from each site were received via a secure electronic data transfer platform and were centrally archived on a project-specific XNAT database [32] at the lead site.

### Site qualification

The site qualification process included a qualitative review of the volunteer (or an exemplar patient) data acquired from each scanner. The image review was performed concomitantly by the radiologist and physicist at the lead site and included a series of elements’ check: correct values of key MR parameters, absence of or minimal artefact/distortion, good fat suppression and sufficient signal-to-noise ratio (SNR), in particular for the high-*b*-value images. Where needed, specific protocol changes were instituted as part of the feedback process to every site. To discuss such additional changes, a virtual conference was organised to brief the site-specific multidisciplinary team and also clarify any unresolved queries. Once all these steps were completed, the site was approved for patient recruitment for the imaging sub-cohort. Additional support (where required) was offered to train radiologists in WB-MRI reporting. This took the form of visits to the lead site or by attending courses organised by the lead site radiologists.

### Initial patient data assessment

Similarly to the site qualification data review requirements, images for the first trial patient scanned at each site were reviewed as well. For sites where no patient was scanned yet, volunteer data were assessed. The cohort included 9 patients and 3 healthy volunteers, 9 men and 3 women with a mean age of 53 years (range 20–72). Almost all patients were scanned prior to the start of treatment, except for two patients that were scanned three months following autologous stem cell transplantation (ASCT).

A radiologist with 3-year experience in WB-MRI qualitatively assessed the DWI (all b values and ADC maps) and T_1_-w Dixon (fat- and water-only maps) images from all stations on a 4-point Likert scale: 1 = non-diagnostic; 2 = sub-optimal; 3 = good; and 4 = excellent. Quantitative assessment of ADC values measured from a 20 cm^2^ region within the bladder fluid was also performed across the same cohort.

## Results

### Protocol

WB-MRI was successfully set up at 12 UK sites involving 3 vendor systems across both 1.5 T and 3 T scanners. The imaging sub-study set up commenced in February 2019, and all 12 sites were opened within a year, with most sites being opened to recruitment by August 2019. Four scanning protocols for 4 different scanners were generated: 1.5 T Siemens (protocol A), 3 T Siemens (protocol B), 1.5 T Philips (protocol C) and 3 T GE (protocol D). Across all sites, data were acquired from 7 scanners of type A, one scanner of type B, 3 scanners of type C and one scanner of type D.

All scanners achieved the desired body scanning coverage from skull vertex to knees within one hour. Core DWI parameters such as recommended *b*-values (at least two mandatory *b*-values: *b* = 50 and *b* = 900 s/mm^2^), large field of view (430 mm), 5 mm slice thickness, minimal echo time (automatically calculated by the scanner) and ability to increase the number of signal averages for the highest *b*-value adhered to published recommendations [10]. The T_1_-w Dixon sequence matched the same anatomical coverage (slice thickness and field of view) as for DWI and was acquired in a single breath hold of < 20 s for each station.

Nonetheless, significant protocol modifications were sometimes made as a result of scanner limitations (both hardware and software) and/or local preferences (see Table 3). Site 9 had a hardware limitation (lower specification of the gradient coil) that led to a longer DWI acquisition that also impeded the SNR of highest *b*-value. To achieve good SNR, the number of signal averages for the highest *b*-value was increased from 6 to 7. Three out of the 12 sites had a limited software performance, which did not include automatic image series composing and required the local team to manually process the images for radiological reading. In addition, due to local preferences, three sites modified their scanning protocol (such as decreasing the number of averages or reducing the number of *b*-values) in order to shorten the DWI acquisition time to fit in with their clinical workflow. Two sites also preferred a slightly larger field of view (450/455 mm instead of 430 mm). Changes of the scanning protocol implemented at each site are summarised in Table 3.

**Table 3:** Factors affecting protocol delivery at each site

Site	Protocol	Hardware limitation	Software limitation	Local preference (protocol changes from Table 2)
1	A	Ok	Ok	–
2	A	Ok	Ok	DWI: fewer averages (2,2,4) to reduce scan time; larger FOV (450 mm) to better cover the arms
3	B	Ok	No station composing	–
4	A	Ok	No station composing	–
5	C	Ok	Ok	–
6	D	Ok	No station composing (DWI only)	–
7	A	Ok	Ok	–
8	C	Ok	Ok	–
9	A	Low gradients (33 mT/m at 125 T/m/s *vs*. 45 mT/m at 200 T/m/s)	Ok	DWI: number of averages for *b* = 900 mm^2^/s was increased to 7 (from 6) to compensate for lower SNR (longer TE; 81 ms) due to lower gradient strength
10	C	Ok	Ok	–
11	A	Ok	Ok	DWI: fewer averages (2,2,4) to reduce scan time; larger FOV (455 mm) to better cover the arms
12	A	Ok	Ok	DWI: only 2 *b*-values (50, 900; omit *b* = 600 mm^2^/s) and fewer averages (2,5) to reduce scan time

FOV field of view; SNR signal-to-noise ratio

### Test objects

**Fig. 2:**
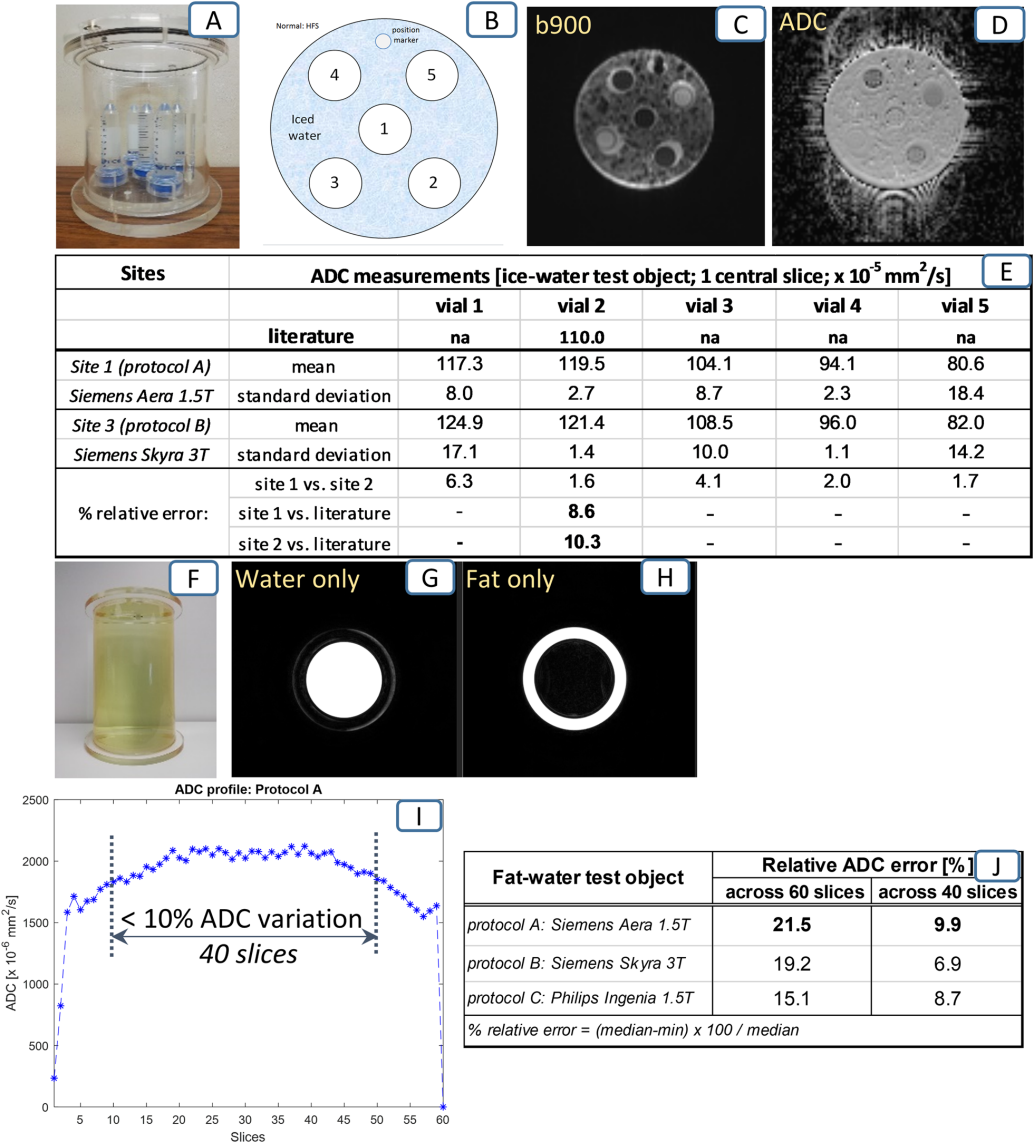
MR techniques’ assessment using the two specialised test objects. Panels **A**–**E**: Temperature-controlled ADC measurements using the ice-water test object. Panels **F**–**J**: Demonstration of good fat suppression technique using the fat–water test object (**F**, **G**, **H**) and good ADC homogeneity along the *z*-direction (**I**, **J**)

Test-object measurements obtained during the two site visits are presented in Fig. 2. ADC values for the five vial components of the ice-water phantom (Fig. 2A–D) were measured in temperature-controlled experiments (ice environment at 0 °C) on a 1.5 T and 3 T scanner, respectively. Compared with literature reference value available for pure water at 0 °C ([33], the maximum ADC relative error observed in our equivalent set-up (vial 2) was 8.6% at 1.5 T and 10.3% at 3 T (highlighted values within table in Fig. 2E). However, the maximum relative error between the measurements over the two sites was lower at 6.3% (range 1.6% to 6.3%, mean 3.2%) across all 5 vials, demonstrating good overall accuracy. Note that our reported ADC relative errors (8.6% and 10.3%; vial 2) are similar to literature reported value of 10%, measured for a pure water vial within an ice-water phantom across 20 scanners [33].

The fat–water phantom experiment showed good and uniform fat suppression on two types of 1.5 T scanner (protocols A and C) and one 3 T scanner (protocol B); examples of images acquired on a Siemens 1.5 T scanner are presented in Fig. 2G, H. Furthermore, the measured ADC profile within the doped water compartment demonstrated homogenous and accurate ADC estimates over a 40 slices (20 cm) coverage in the cranio-caudal direction, as shown in Fig. 2I (variation less than 10% from central slice). However, note that a larger cranio-caudal coverage (of 41–60 slices) would degrade ADC measurements by as much as 22% variation. Equivalent values were found for the Philips 1.5 T scanner (protocol C) and the Siemens 3 T scanner (protocol B), see table in Fig. 2J. Therefore, we suggest using a number of 40 slices per each DWI station, in agreement with previous recommendations [27].

### Volunteer data

**Fig. 3:**
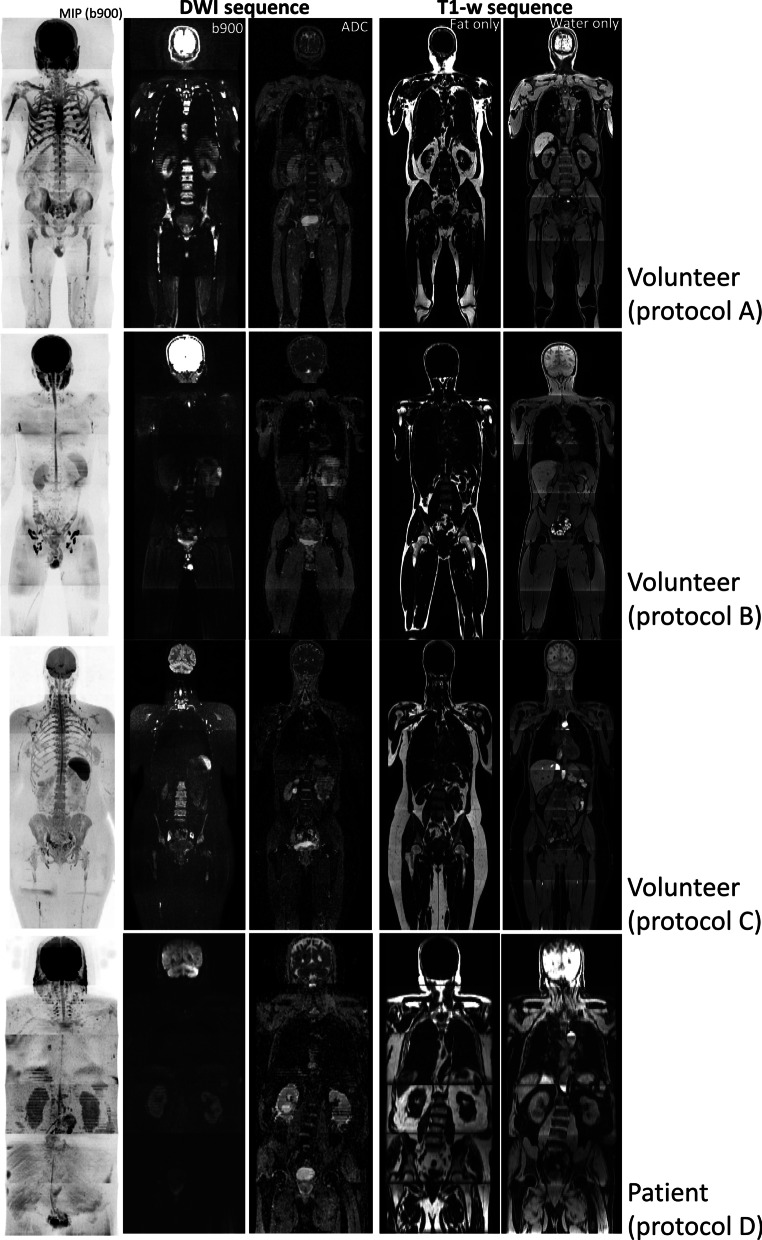
Volunteer/patient images acquired using the four main MR protocols. Coronal reconstructions showing composed MIP, b900 and ADC images (for DWI sequence) and composed fat- and water-only images (for T_1_-w Dixon sequence) for protocol A (Siemens 1.5 T), B (Siemens 3 T), C (Philips 1.5 T) and D (GE 3 T)

Coronal reconstruction of multi-station axial acquisitions allows visualisation of all scanned stations in one go. Examples of DWI coronal reconstructions for healthy volunteers (protocols A–C) and a patient (protocol D) are presented in Fig. 3. Typical example of DW images (b900, ADC map) and MIP reconstruction for the b900 images demonstrates good overall quality across the 4 protocols. Qualitative radiological assessment confirmed appropriate image SNR, uniformity of fat suppression, minimal image distortion and sufficient bone marrow visualisation. Similarly, good image quality was also observed for the corresponding T_1_-w images (including the water only and fat only).

**Fig. 4:**
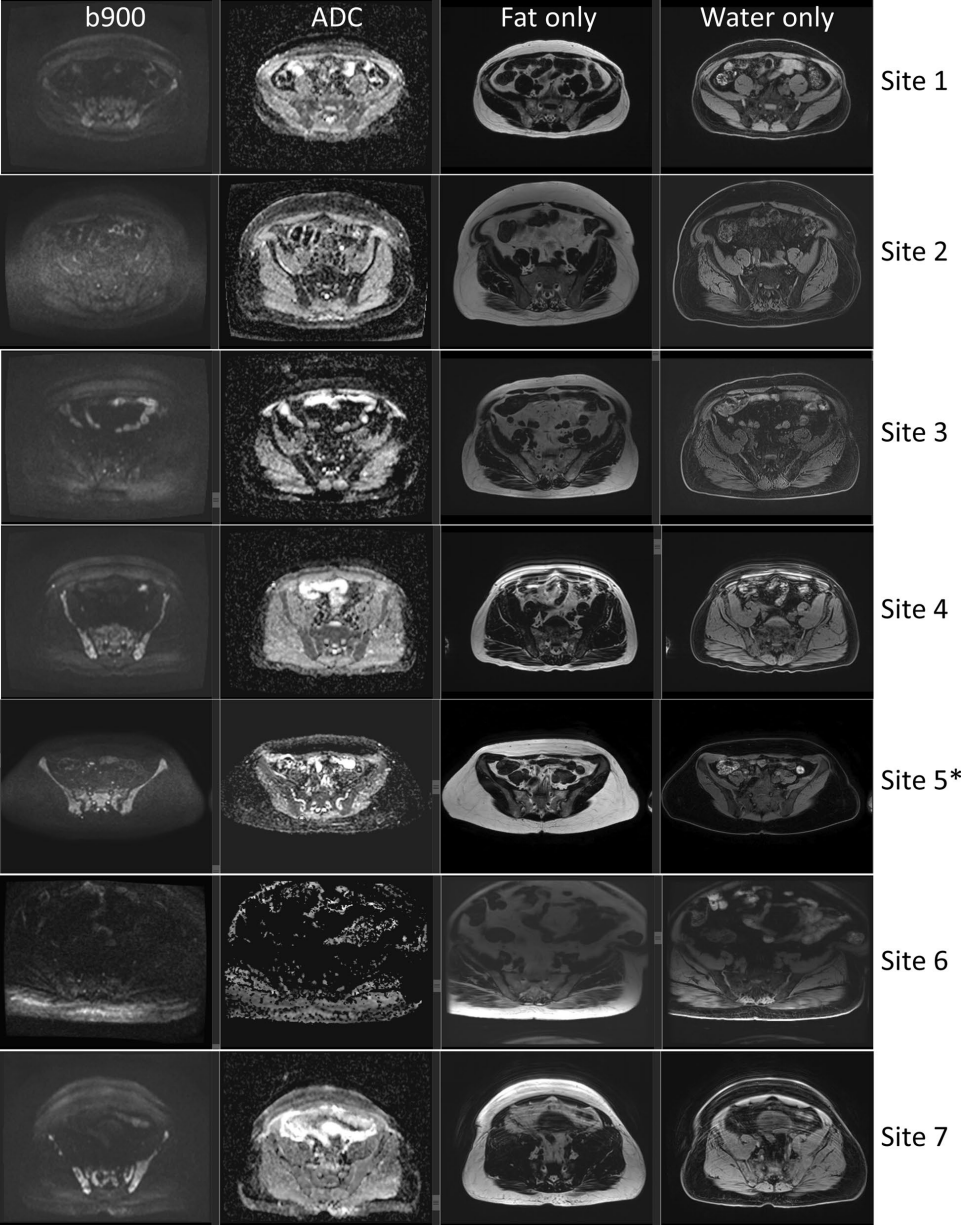

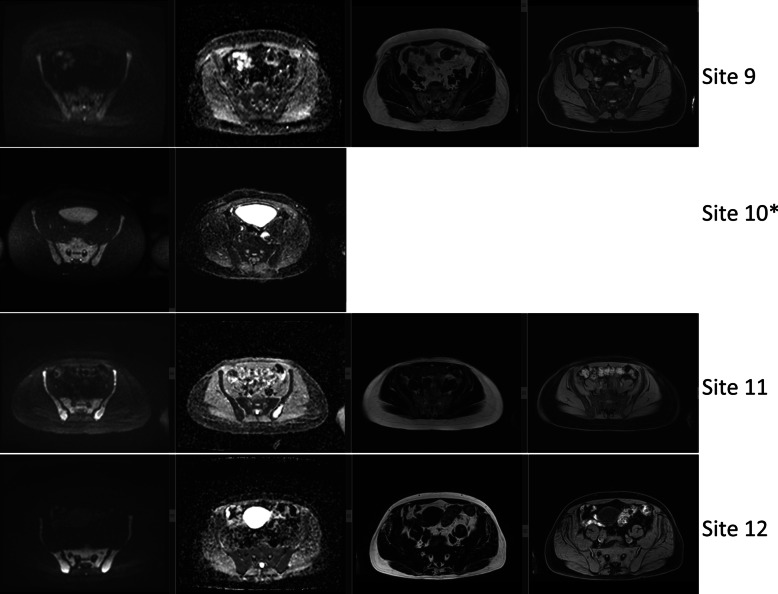
Initial MR images from each site. Axial images through the pelvis for DWI (b900, ADC) and T_1_-w Dixon (fat, water) sequences acquired on the first datasets (9 patients and 2 volunteers) from each site; lines correspond to scanner information listed in Table 1. No data are presented for site 8 as data were unavailable (incomplete). Volunteer data are marked by the * sign

Figure 4 shows the extent of variability in the DWI and T_1_-w images acquired at each site. Note that the example patient scanned using protocol D demonstrated unacceptable DWI quality of axial high-*b*-value (b = 900 s/mm^2^) images in the pelvis area (line 6 in Fig. 4), although the coronal reconstruction was deemed to be acceptable (line 4 in Fig. 3). To counteract this lower DWI quality, a series of protocol changes were made and implemented at the site before the trial recruitment commenced; note that Table 2 presents final MR parameters amended for protocol D.

### Patient data

Table 4 presents the qualitative radiological assessment of the DWI and T_1_-w Dixon images for initial patients acquired at 9 sites and 3 volunteers scanned at sites that have not yet commenced patient recruitment. The following images were assessed across all stations: all *b*-values and the ADC map for DWI and the fat- and water-only images for Dixon sequences. Across the 12 sites, eight sites received a maximal overall score of 4 (excellent), two sites were considered level 3 (good) and one site (site 6) was scored sub-optimal in particular for the DWI quality. Unfortunately, data from site 8 were incomplete and a full assessment was not possible. Specific comments for each site are detailed in Table 4.

**Table 4:** Radiologi﻿cal assessment of image quality of initial data (patient or volunteer) from each centre

Site	Protocol	Images	Pat/Vol	Sequence score	Overall scan score	Radiologist comments
1	A	DWI	p	4	4	Ok
		Dixon	p	4		Ok
2	A	DWI	p	4	4	Susceptibility artefact anterior thoracic
		Dixon	p	4		Minor fat/water swap; susceptibility artefact anterior thoracic
3	B	DWI	p	2	3	Eddy current, ghosting, B1 artefact, anterior thoracic signal loss
		Dixon	p	4		Minor fat/water swap
4	A	DWI	p	4	4	Loss of signal in liver & anterior thighs
		Dixon	p	4		Minor breathing artefact tummy
5	C	DWI	v	4	4	Ok
		Dixon	v	4		Ok
6	D	DWI	p*	1	2	ADC non-diagnostic, noisy DWI, large patient
		Dixon	p*	3		Extensive fat/water swaps, large patient
7	A	DWI	p	3	3	Breathing artefact (but cardiac failure with bilateral effusions and important soft tissue oedema)
		Dixon	p	3		
8	C	DWI	v	n/a	n/a	DWI data unavailable
		Dixon	v	4		Incomplete Dixon data
9	A	DWI	p	3	4	Blurred b900, especially for ribs, humeri and proximal femora
		Dixon	p	4		Ok
10	C	DWI	v	4	4	Ok
		Dixon	v	4		Dixon data unavailable for pelvic region
11	A	DWI	p	4	4 –	Ok
		Dixon	p	3		"Average slab" effect
12	A	DWI	p	4	4	Minor loss of signal anteriorly chest & abdomen
		Dixon	p	4		Ok

DWI (all b values and ADC maps) and T_1_-w Dixon (fat- and water-only maps) images from all stations were assessed radiologically on a 4-point Likert scale: 1 = non-diagnostic; 2 = sub-optimal; 3 = good; 4 = excellent

^*^ Example of clinical patient for MR protocol testing (patient not within trial)

Table 5 summarises quantitative assessment of the DWI data across the 12 subjects. A mean ADC value of 309 × 10^–5^ mm^2^/s (standard deviation 29.5, range 271–375) was found for the bladder fluid. A maximal relative difference of ADC (versus the mean cohort value) of 5% was observed between 3 sites using identical scanners and protocols (sites 1, 4 and 7). No strong deviation from the cohort ADC could be attributed to a particular vendor or a specific type of scanner.

**Table 5:** Quantitative assessment of initial human data across all sites (same cohort as for Table 4): measurements of ADC values of the bladder fluid

Site	Protocol	Subject	Gender	Age	Disease status	ADC (20cm^2^ROI within the bladder)		
				Years		Mean	st dev	% relative difference
1	A	p	m	56	Untreated	326	16	5
2	A	p	m	64	Treated	333	22	8
3	B	p	m	68	Untreated	285	15	− 8
4	A	p	m	59	Untreated	311	18	1
5	C	v	f	31	Healthy volunteer	375	19	21
6	D	p*	m	72	Unknown	295	18	− 4
7	A	p	m	45	Untreated	295	20	− 4
8	C	v	f	29	Healthy volunteer	na	na	na
9	A	p	m	71	Untreated	292	17	− 6
10	C	v	m	20	Healthy volunteer	327	14	6
11	A	p	f	55	Treated	271	29	− 12
12	A	p	m	66	Untreated	286	24	− 7
**Cohort mean values**				**53**		**309**	**19**	**0**

Demographics (gender, age and disease status) for each subject is included. The relative difference was measured against the mean cohort ADC value

p = patient; v = volunteer; p* = non-trial patient

m = male; f = female

na = not available

treated = 3 months post-ASCT (autologous stem cel transplantation)

ADC = apparent diffusion coefficient; units of 10^-5^mm^2^/s; ROI = region of interest

## Discussion

Our analysis shows that following a careful quality control and quality assurance process, high-quality whole-body MR images can be achieved using scanners from three main manufacturers at two different field strengths, across a wide range of sites within the UK. We were able to implement, at all sites, protocols that were compliant with the MY-RADS guidelines and the resulting data were suitable for both qualitative and quantitative analysis. Our prior experience of WB-MRI on similar platforms helped to minimise time and resource burden when helping to set up a WB-MRI protocol at a new site, for example, by transferring validated protocols, which could be directly loaded onto the scanner.

WB-MRI, including DW-MRI, has been used in multi-centre studies for other diseases, e.g. lymphoma [34, 35], paediatric Hodgkin lymphoma [36], colorectal and lung cancer [37, 38], and also in healthy volunteers [39], demonstrating that WB-MRI is an established technique that is suitable for use in multi-centre deployment. Despite its application elsewhere, the use of WB-MRI has been limited in multi-centre studies for myeloma. As far as we are aware, our current trial represents the first imaging sub-study to employ a standardised WB-MRI protocol within a prospective multi-centre clinical trial in myeloma in the UK.

Most multi-centre WB-MRI studies have been limited to using identical scanners [39] or by limiting the range of manufacturers or field strengths included. For example, one study of 108 patients with lymphoma was carried out by two 1.5 T scanners from two manufacturers and matched the main imaging protocol (i.e. DWI and standard T1-w) parameters between the two scanners [34]. Another study of 26 patients with lymphoma used four 1.5 T scanners, including three different manufacturers [35], also matching the main imaging protocol parameters between the scanners. Another study of 68 paediatric patients with Hodgkin lymphoma used ten 1.5 T scanners, including three different manufacturers; some of the main imaging protocol parameters were matched between scanners, with a wider range allowed for some of the sequences [36]. Yet some other multi-centre WB-MRI studies have taken a pragmatic approach by specifying a small number of key parameters, such as anatomical coverage, slice thickness and *b*-values for DW-MRI, whilst allowing sites to set up their own imaging protocols [37, 38]. Such an approach may facilitate rapid set-up of a multi-centre study, but may limit acquisition of imaging data to sites with existing WB-MRI expertise or run the risk of some sites returning images of poorer quality.

Note that typically the WB-MRI main protocol included axial DWI and coronal T_1_-w and T_2_-w imaging [35, 36]. More recently, the WB-MRI protocol shifted towards matching axially the DWI and T_1_-w Dixon sequences [26, 38] to allow a direct use of derived parametric maps such as ADC (from DWI) and relative fat fraction (from T_1_-w Dixon).

By carring out this study, we demonstrated the feasibility of setting up a multi-centre WB-MRI study, including matched DW-MRI and T_1_-w Dixon MRI sequences, with a high degree of standardisation, within a relatively short time period. This was also achieved without extensive use of scanner time at external sites for protocol set-up. Providing our imaging protocols in sufficient detail (see Table 2) can help other sites to replicate our protocols on their similar MR systems to yield operationally ready MY-RADS-compliant WB-MRI protocols. Protocols can be implemented on widely available scanners at no further costs or software requirements.

A high degree of alignment and standardisation in scan protocols is also advantageous for future quantitative use of data. Whilst the primary endpoints of many WB-MRI studies are reliant on visual interpretation of images by radiologists, who are able to cognitively account for signal variations arising from the imaging protocols [35–38], it is important to note that large multi-centre datasets are valuable resources that can be harnessed for additional exploratory analysis. Previous observations have highlighted the challenges of using heterogeneous data sets, for example retrospective use of multi-centre WB-MRI data in machine learning applications [40]. Hence, the acquisition of prospective multi-centre WB-MRI data with a high degree of standardisation can enhance the scope to make use of such data for further analysis after conclusion of the main study.

Although highly encouraging, our report shows several limitations in our approach towards standardisation. Protocol changes requiring modification of scan parameters were encountered in three settings: hardware (e.g. lower-specification gradient coils), software (e.g. absence of multi-station composing packages) and time constraints. Whilst the first limitation cannot be easily overcome, one could consider how best to address the time issue.

Typically, a complete WB-MRI protocol requires up to 1 h scanning time, which may be considered too long for sites with limited MR capacity. Hence, for 3/12 sites (sites 2, 11 and 12), a protocol with shorter acquisition time (i.e. fewer averages), but with minimal impact on DWI quality and ADC calculations (Table 5), was advocated. ADC estimate obtained by site 12 whilst using only 2 *b* values (from the recommended three) was similar to the cohort mean ADC (286 vs. 309 × 10^–5^ mm^2^/s); this result was in agreement with a recent literature report [41] that compared breast ADC estimates derived from 2 (instead of 4) b values and found no impact on the MRI diagnostic performance. Overall, the slight protocol adaptations and inter-vendor or inter-centre variability generated a maximal relative difference of ADC of 21%. For future trials, where absolute values of ADC might be used as a patient stratification tool for either diagnostic or treatment response, there is an imperative need for rigorous standardisation of WB-DWI protocols.

The limitations of unavailable software for inline image composing was solved by generating an in-house script that was able to compose DWI and T_1_-w Dixon maps from different manufacturers at the lead site when these images were collected.

One of the lessons learnt was that the time available for protocol optimisation at external sites was usually limited compared with the time available to undertake the protocol development at the lead site. Hence, it would be important to be fully acquainted with the full protocol on various scanners to facilitate the technology transfer. Nonetheless, it is worth noting that the optimum parameters or methodology available on one MRI platform may not be available or achievable on another. For this reason, it is important to work with the site-specific team who are more familiar with local scanner to come to a creative and agreeable solution; otherwise, the general protocol should be modified to omit elements that cannot be easily achieved by all scanners. For example, the relative fat fraction maps have recently been recommended [26] to be used in conjunction with the DWI and ADC maps for a better diagnosis and assessment of treatment response for bone disease. At the time of setting up this trial, some scanners were not able to automatically generate the relative water and fat fraction maps; therefore, such an output map was not sought explicitly as part of this trial set-up.

Attention should also be paid to the fact that a complete MR dataset encompassing both acquired and post-processed/composed datasets can be very large (~ 3 GB) which could impact on data transfer. Due to their size, the transfer of images may be delayed due to the uploading and downloading steps at each site. As part of the QA/QC process, the success and rapidity of data transfer between the trial sites should also be verified.

As WB-MRI is increasingly used for different clinical scenarios, and not only for myeloma, further technical and software developments are progressively being implemented by the MRI vendors. This would undoubtedly facilitate the inclusion of common features in multi-centre trials using WB-MRI.

## Conclusions

We demonstrate that standardised MY-RADS WB-MRI protocols can be implemented and supported in prospective multi-centre clinical trials at sites with limited prior experience of WB-MRI. WB-MRI is not a technology that has to be restricted mainly to MRI research centres. This methodology enables imaging research to follow patient need and to be undertaken in high-recruiting centres, allowing other sites a reduced burden on time and resources.

## Data Availability

Due to privacy regulations, the data used in this study are not publicly available. In order to see and discuss the data, the authors can be contacted. If needed, we can arrange approval to share the data with individual researchers.

## Abbreviations

ADC: Apparent diffusion coefficient
ASCT: Autologous stem cell transplantation
DWI: Diffusion-weighted imaging
DW-MRI: Diffusion-weighted MRI
FDG: Fluorodeoxyglucose
IMAGIMM: IMAGIng minimal residual disease in myeloma
IMWG: International myeloma working group
MET-RADS-P: METastasis reporting and data system for prostate
MIP: Maximum intensity projection
MRD: Minimal residual disease
MY-RADS: Myeloma response assessment and diagnosis system
ONCO-RADS: Oncologically relevant findings reporting and data system
PET/CT: Positron emission tomography/computed tomography
QA: Quality assurance
QC: Quality control
SNR: Signal-to-noise ratio
T1-w: T1-weighted
WB-MRI: Whole-body MRI
